# Human immunosuppressive neutrophils: recent answers to old and new questions

**DOI:** 10.1038/s44318-026-00829-6

**Published:** 2026-06-05

**Authors:** Patrizia Scapini, Chiara Lattanzi, Sven Brandau, Marco A Cassatella

**Affiliations:** 1 Department of Medicine, Section of General Pathology, University of Verona, Verona, 37134 Italy; 2 Research Division, Department of Otorhinolaryngology, University Hospital Essen, Essen, 45122 Germany; 3 German Cancer Consortium, Partner Site Essen-Düsseldorf, Essen, Germany

**Keywords:** Immunology

## Abstract

Polymorphonuclear myeloid-derived suppressor cells (PMN-MDSCs) represent the most investigated type of “immunosuppressive” neutrophils in humans. However, a detailed understanding of human PMN-MDSCs has been hindered for years by limited knowledge of their specific markers for sorting and purification, as well as by their very low numbers in patient circulation. Recently, the use of advanced technologies has shown that human PMN-MDSCs are generated during emergency granulopoiesis and comprise a heterogeneous population of both immature and mature neutrophils. It has also been shown that human mature PMN-MDSCs (**m**PMN-MDSCs) perform immunosuppressive functions through transcriptional reprogramming during maturation. Furthermore, single-cell RNA sequencing experiments have shown that human **m**PMN-MDSCs and tumor-associated neutrophils (TANs) share transcriptomic similarities, therefore uncovering a potential relationship between them. In this Perspective, we discuss: i) how our knowledge on human PMN-MDSCs has advanced; ii) the criteria for discriminating human PMN-MDSCs from normal neutrophils or other immunosuppressive neutrophil populations; iii) recent findings revealing that human mPMN-MDSCs exhibit transcriptomic features suggestive of functions beyond immunosuppression.

## Abstract (web summary)

This perspective discusses recent insights into the origins, specific features and disease-associated functions of human polymorphonuclear myeloid-derived suppressor cells.

## Graphical abstract

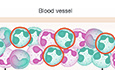

## Introduction

**Fig. 1:**
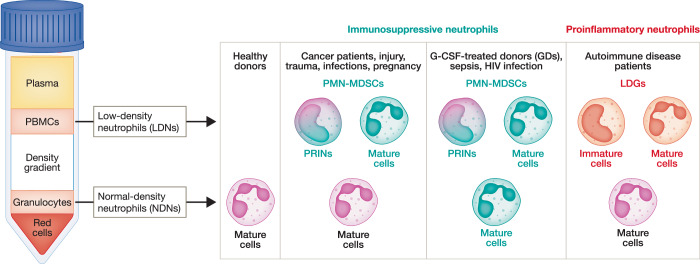
Cartoon displaying the main populations of immunosuppressive and proinflammatory neutrophils included within LDNs and NDNs. After centrifugation of blood from healthy donors over Ficoll-Paque density gradients, a homogeneous population of resting granulocytes, including normal-density neutrophils (NDNs), typically sediment over the red cell pellet. By contrast, density gradient centrifugation of blood from patients with acute and chronic inflammatory diseases manifests the presence of so-called low-density neutrophils (LDNs) within the mononuclear cell fraction, in addition to NDNs. LDNs consist of a mixture of immature neutrophils (including band cells, metamyelocytes, myelocytes and promyelocytes) and mature neutrophils at variable ratios, which, depending on the disease, may display either immunosuppressive or proinflammatory properties. Immunosuppressive LDNs are also known as polymorphonuclear myeloid-derived suppressor cells (PMN-MDSCs) and have been mostly characterized in cancer, but are also found in infections, trauma, as well as in pregnant women and in G-CSF-treated donors (GDs). PMN-MDSC progenitors undergo a complex transcriptional reprogramming during their differentiation, which is already evident at the level of the generated immature neutrophils [i.e., PMN-MDSC-reprogrammed immature neutrophils (PRINs)]. PRINs ultimately mature into mature PMN-MDSCs (**m**PMN-MDSCs), which represent the most effective immunosuppressive component of PMN-MDSCs. Under conditions characterized by severe systemic inflammation—such as those occurring in patients with sepsis, HIV or in GDs—the emergency granulopoiesis-mediated reprogramming becomes so accelerated that even the NDNs, in addition to LDNs, acquire the phenotypic and functional features of **m**PMN-MDSCs. By contrast, proinflammatory LDNs, known as low-density granulocytes (LDGs), are instead found in systemic lupus erythematosus (SLE), psoriasis, chronic granulomatous disease (CGD), and antineutrophil cytoplasmic antibody-associated vasculitis (AAV).

Polymorphonuclear neutrophil populations that, once purified from the blood or tissue, prove able to suppress in vitro T cell proliferation and/or T cell activation (i.e., production of IFNγ) are defined as “immunosuppressive” (Aarts and Kuijpers, 2018; Antuamwine et al, 2023; Grover et al, 2025; Pillay et al, 2013; Scapini et al, 2016). Among them, the so-called PMN-myeloid-derived suppressor cells (PMN-MDSCs) [also known as granulocyte-MDSCs (G-MDSCs) (Antuamwine et al, 2023; Grover et al, 2025; Talmadge and Gabrilovich, 2013) or neutrophil-MDSCs (N-MDSCs) (Koenderman and Vrisekoop, 2025)] represent the most investigated type of “immunosuppressive” neutrophils in humans. Human PMN-MDSCs, commonly considered to correspond to the neutrophilic cells known as low-density neutrophils (LDNs), which abnormally sediment within peripheral blood mononuclear cells (PBMCs) after blood density centrifugation (Fig. 1; Box 1), are indeed detectable in patients with cancer (Antuamwine et al, 2023; Scapini et al, 2016; Talmadge and Gabrilovich, 2013; Zea et al, 2005) and other conditions. The latter includes sepsis (Charoensappakit et al, 2025; Darcy et al, 2014; Janols et al, 2014; Sun et al, 2022; Uhel et al, 2017), viral infections (Bowers et al, 2014; Coudereau et al, 2022; Vollbrecht et al, 2012), severe injury and trauma (Bryk et al, 2010), neonates (Rieber et al, 2013), pregnant women (Kostlin et al, 2016; Li et al, 2020), as well as healthy donors (HDs) subjected to G-CSF administration (named as GDs) for transplantation purposes (Luyckx et al, 2012; Marini et al, 2017; Pettinella et al, 2024; Tumino et al, 2020; Vasconcelos et al, 2003). However, to date, most research on human PMN-MDSCs primarily focuses on their involvement in cancer, as their frequencies—usually ranging between 1 and 10% of total PBMCs—have been demonstrated to negatively correlate with tumor stage, prognosis, and therapeutic response (Bergenfelz and Leandersson, 2020; Scapini et al, 2016; Talmadge and Gabrilovich, 2013). Several factors have, for years, impeded a more rapid and detailed molecular characterization of human PMN-MDSCs, including their very low numbers, their heterogeneous composition of mature and immature cells, and the lack of specific markers. Nonetheless, given advances in technology, recent research has provided a more detailed characterization of human PMN-MDSCs, including their origin, composition, transcriptomes, phenotypes, and functions. The new question is how PMN-MDSCs relate to the plethora of other types of neutrophils performing immunosuppressive activities, including the circulating CD62L^dim^CD16^bright^ neutrophils identified in subjects injected with low doses of endotoxin or with severe injury (Pillay et al, 2012), or to the normal-density neutrophils (NDNs) isolated from HDs and shown to acquire immunosuppressive states upon incubation in vitro with appropriate cues (see below).

Hence, in this Perspective, we will not only discuss how our knowledge on human PMN-MDSCs has advanced, but we will also address the issue concerning the relationship between human PMN-MDSCs and the other types of “immunosuppressive” neutrophils, with the purpose of providing the bases for solid criteria usable to create a correct classification and/or nomenclature of these cells. Although the knowledge of murine PMN-MDSCs is more extensive than that of their human counterparts, we will not assume that murine and human PMN-MDSCs are identical. We will therefore refer to murine PMN-MDSCs only when appropriate. In fact, there are fundamental differences between human and murine neutrophil/PMN-MDSC biology that have a profound impact on their intrinsic features and functions (Eruslanov et al, 2017; Nauseef, 2023). We will mainly (but not exclusively) discuss PMN-MDSCs from either cancer patients or GDs, since they are the most thoroughly characterized at the molecular level. For an immediate comprehension of the text, from now on the terms: (i) “PMN-MDSCs” will refer to the total circulating CD66b^+^ PMN-MDSCs (i.e., including mature and immature neutrophils); (ii) **m**PMN-MDSCs will indicate the mature CD66^+^CD11b^+^CD16^+^CD10^+^ cell fraction of PMN-MDSCs, while PRINs will indicate their “PMN-MDSC-reprogrammed immature CD66^+^CD11b^+/-^CD16^+/−^CD10^−^ neutrophil fraction” (ranging from the promyelocyte to the band stage); (iii) NDNs will refer to the non-suppressive CD66^+^CD11b^+^CD16^+^CD10^+^mature neutrophils from HDs, unless differently specified.

Box 1 Human PMN-MDSCs The term MDSCs was first introduced in 2007 (Gabrilovich et al, 2007) to describe populations of immunosuppressive myeloid cells at various stages of differentiation, including both monocytic (Mo-MDSCs) and granulocytic (PMN-MDSCs) subsets, found in numerous pathological conditions of both humans and mice (Antuamwine et al, 2023; Grover et al, 2025; Scapini et al, 2016). Human PMN-MDSCs consist of a mixture of mature and immature cells typically corresponding to the “low-density neutrophils (LDNs)”, so named because, upon blood centrifugation on density gradients, they sediment within the layer of PBMCs due to their lower density compared with that of normal mature neutrophils (i.e., normal-density neutrophils, NDNs; Fig. 1) (Bergenfelz and Leandersson, 2020; Scapini et al, 2016). It is, however, necessary to recall that the detection of LDNs in the blood of human subjects does not automatically imply that they exert “immunosuppressive” activities and that, therefore, qualify for the “PMN-MDSC” term. For instance, LDNs are generally either absent or present at levels below 0.5 - 1% of total PBMCs in the blood of healthy donors (HDs). However, NDNs may artificially decrease their buoyancy and consequently accumulate within the PBMCs, either because they may degranulate in vivo (Aarts et al, 2019; Aarts and Kuijpers, 2018), or as an artifact of improper blood handling during non-standardized leukocyte isolation techniques (Cassetta et al, 2020). The choice of anticoagulant, the type of density gradient, and the time elapsed between blood collection and processing are indeed all factors that can lead to an increased recovery of LDNs. However, these conditions lead to the generation of activated LDNs, which kill rather than suppress T cells (Aarts et al, 2019; Aarts and Kuijpers, 2018; Blanco-Camarillo et al, 2021) and even B cells (Lelis et al, 2017). In this context, the work by Cassetta L. and colleagues (Cassetta et al, 2020) outlines the protocols for the correct processing of blood samples, enabling the accurate study of PMN-MDSCs. Moreover, LDNs are also present in autoimmune pathologies [such as, for example, systemic lupus erythematosus (SLE), rheumatoid arthritis, and psoriasis], in which they have been arbitrarily named as low-density granulocytes (LDGs) since they have been shown to display enhanced proinflammatory functions ex vivo (i.e., NETtosis, cytokine production, proinflammatory mediators release, etc.) (Carmona-Rivera and Kaplan, 2023), other than activating (rather than suppressing) T cells (Marini et al, 2017; Rahman et al, 2019) (Fig. 1). Therefore, LDNs must always be functionally tested ex vivo to clarify whether they function as immunosuppressive (i.e., PMN-MDSCs) or as proinflammatory (i.e., LDGs) cells. Performing standardized T cell-immunosuppression assays to evaluate the natural PMN-MDSC ability to induce T cell anergy rather than T cell killing is also crucial for obtaining reliable data that can be compared across laboratories, thereby avoiding controversial results. The review by Bruger A.M. and colleagues (Bruger et al, 2019) exhaustively recapitulates and critically discusses the various types of experimental assays used to investigate neutrophil-mediated T cell immunosuppression.

## On the origin of human PMN-MDSCs

One of the long-standing questions regarding PMN-MDSCs in humans is whether they originate from BM precursors undergoing an altered process of granulopoiesis (Aliazis et al, 2024; Bergenfelz and Leandersson, 2020; Grover et al, 2025; Pillay et al, 2013; Scapini et al, 2016), or from circulating mature neutrophils that acquire immunosuppressive properties in response to disease-specific factors (Rodriguez et al, 2009; Schmielau and Finn, 2001; Sippel et al, 2011). Although never formally demonstrated in humans, the general assumption based on mouse data (Grover et al, 2025) is that PMN-MDSCs are mobilized from the  bone marrow (BM) in response to etiologic factors causing an “emergency granulopoiesis” response (Aliazis et al, 2024). Emergency granulopoiesis is the process of accelerated, de novo production and mobilization of granulocytes (primarily neutrophils) from the BM [or other extramedullary organs, such as the spleen (Cole et al, 2021; Guo et al, 2025; Jordan et al, 2017; Tavukcuoglu et al, 2020)] to meet an overwhelming leukocyte demand caused by an insult (Manz and Boettcher, 2014). An emergency granulopoiesis-dependent mobilization of human PMN-MDSCs is also consistent with their mixed composition of immature/mature cells (Antuamwine et al, 2023; Bergenfelz and Leandersson, 2020; Scapini et al, 2016). Some attempts to generate PMN-MDSCs in vitro from immature precursors, mimicking the conditions occurring during an “emergency granulopoiesis-like” process, have been performed, for instance, by incubating CD34^+^ hematopoietic progenitors isolated from various sources [i.e., HD-BM aspirates (Marigo et al, 2010; Solito et al, 2011), cord blood from pregnant women (Park et al, 2019), or peripheral blood of GDs (Casacuberta-Serra et al, 2017)] with different cytokine/hematopoietic growth factor cocktails, such as SCF, TPO, FLT3-L, GM-CSF, G-CSF, IL-6, and/or IL-3. Even HD PBMCs [known to contain hematopoietic stem and progenitor cells (HSPCs), common myeloid progenitors (CMPs), and granulocyte-monocyte progenitors (GMPs) (Quaranta et al, 2024)] cultured in the presence of various cytokine/hematopoietic growth factor cocktails, including GM-CSF, IL-1β, PGE2, TNF, or VEGF, have been utilized to generate putative PMN-MDSCs in vitro (Gorgun et al, 2013; Krumm et al, 2023; Lechner et al, 2010; Singh and Rieber, 2021). However, the cell output described in the aforementioned studies primarily consisted of a mixture of myeloid cells at various stages of maturation, exhibiting immunosuppressive capacity. In addition, they were neither thoroughly characterized with respect to their immunosuppressive mechanisms nor at the transcriptomic and/or phenotypic levels. Consequently, it is not possible to assess whether the cells generated in these studies perfectly trace the distinctive transcriptomic, phenotypic, and functional features of **m**PMN-MDSCs and/or PRINs that have been subsequently delineated (see next paragraphs). In this context, the recent discovery and availability of very immature CD34^+^neutrophil-committed progenitors (NCPs) (Calzetti et al, 2022; Signoretto et al, 2025), which retain a substantially greater proliferative and expansion capacity than more mature CD34^⁻^neutrophil precursors, and which - unlike CD34^+^HSCs - exclusively generate neutrophils, point to new cellular candidates suitable for ex vivo manipulation studies to promote the generation of PMN-MDSCs. However, it is evident that achieving this goal requires a more detailed understanding of the molecular mechanisms underlying the reprogramming into PMN-MDSCs occurring during an “emergency granulopoiesis” process.

## Differences between human PMN-MDSCs and putative immunosuppressive neutrophils

Several studies have reported the acquisition of “immunosuppressive states” (Quail et al, 2022) by NDNs “activated” in vitro for adequate periods of time with a variety of cues, such as tumor-derived supernatants (Dahal et al, 2024)/tumor-ascites fluid supernatants (Emmons et al, 2021), serum from cancer patients (Choi et al, 2012), phorbol esters (PMA) (Marini et al, 2017; Rodriguez et al, 2009), tumor necrosis factor (TNF) (Aarts et al, 2019; Rotondo et al, 2011), ionomycin (Rotondo et al, 2011), N-formylmethionyl-leucyl-phenylalanine (fMLP) (Aarts et al, 2019; Rodriguez et al, 2009; Schmielau and Finn, 2001), lipopolysaccharide (LPS) (Aarts et al, 2019), zymosan (Hock et al, 2012), thapsigargin (THG) or dithiothreitol (DTT) - two endoplasmic reticulum (ER) stress inducers (Condamine et al, 2016; Nan et al, 2018), hypoxia (Chang et al, 2025), decidual explant supernatant (Li et al, 2020), and more. However, a major issue to consider concerning these in vitro-activated NDNs is that the underlying suppressive mechanism of the inhibition was either not demonstrated (Chang et al, 2025; Condamine et al, 2016; Dahal et al, 2024; Nan et al, 2018), or (in several studies) shown to depend on mechanisms that ultimately kill T cells, such as the release of elevated amounts of reactive oxygen species (ROS) (Aarts et al, 2019; Choi et al, 2012; Hock et al, 2012; Marini et al, 2017; Schmielau and Finn, 2001; Singel et al, 2019), or trogocytosis (Emmons et al, 2021), while PMN-MDSCs suppress T cell mainly *via* their arginase-1 (ARG-1) release (Antuamwine et al, 2023; Lang et al, 2018; Scapini et al, 2016; Zea et al, 2005). It should also be noted that in vitro-activated NDNs that acquire immunosuppressive properties are often arbitrarily named PMN-MDSCs simply because of their putative immunosuppressive action (Aarts and Kuijpers, 2018; Condamine et al, 2016; Dahal et al, 2024), or because they are found to release pre-stored ARG-1 into the culture medium, in turn accounting for the observed suppressive activity (Rodriguez et al, 2009; Rotondo et al, 2011; Sippel et al, 2011). Naming any type of suppressive neutrophils as PMN-MDSCs is improper, not only because it is adopted without a prior careful evaluation of PMN-MDSC phenotypic, transcriptomic, and functional characteristics, but especially with respect to fundamental aspects and implications of the immunosuppressive action exerted by PMN-MDSCs. Unlike  in vitro-generated immunosuppressive NDNs from HDs, PMN-MDSCs exert a natural suppressive effect on both CD4⁺ and CD8⁺T cell proliferation and activation when freshly isolated from blood and directly co-cultured with T cells, without undergoing any in vitro pre-activation. Ultimately, “bona fide” PMN-MDSCs induce a T cell anergic state promoted by ARG-1 release rather than T cell death (Antuamwine et al, 2023; Bergenfelz and Leandersson, 2020; Scapini et al, 2016; Talmadge and Gabrilovich, 2013). PMN-MDSCs, in fact, accumulate high levels of ARG-1 mRNA and consequently of ARG-1 protein (Cane et al, 2025; Lang et al, 2018; Marini et al, 2017; Rodriguez-Ubreva et al, 2017; Zea et al, 2005), which results from a complex transcriptional reprogramming that occurs during their differentiation (see below). By contrast, HD NDNs store a fixed amount of ARG-1 protein in their granules during normal maturation but downregulate ARG-1 mRNA, thereby becoming unable to replenish it thereafter (Cane et al, 2025; Jacobsen et al, 2007; Munder et al, 2005). It is therefore evident that  in vitro-activated NDNs that kill T cells cannot be considered as “bona fide” PMN-MDSCs. Even though we retain as unlikely the possibility that NDNs could be reprogrammed in vitro to become “bona fide” PMN-MDSCs, it cannot be excluded with certainty that it may occur upon discrete experimental conditions. Notably, human tumor-associated neutrophils (TANs) have also been demonstrated to display elevated expression of ARG-1 mRNA (Cane et al, 2025; Si et al, 2019; Ugolini et al, 2020), as well as to express (Si et al, 2019; Ugolini et al, 2020) and release (Rotondo et al, 2009) high levels of ARG-1 protein. These observations suggest that the maintenance of elevated ARG-1 mRNA/protein expression by PMN-MDSCs and TANs represents a relevant mechanism for sustaining ARG-1-mediated immunosuppression within the blood and the TME, respectively.

Although evidence exists for the use of additional immunosuppressive mechanisms beyond ARG-1 by PMN-MDSCs, the supporting data are not as robust. For instance, PMN-MDSCs from breast cancer patients have been shown to inhibit T cell functions *via* a ROS-dependent mechanism (Mehmeti-Ajradini et al, 2020), while PMN-MDSCs from head and neck cancer (HNC) (Condamine et al, 2016) or hepatocellular carcinoma (Nan et al, 2018) patients have been shown to rely on both ROS and ARG-1 release. However, more recently, using specific inhibitors, PMN-MDSCs from HNC have been shown to rely on ARG-1, but not on ROS or NO release (Lang et al, 2018). Nonetheless, human PMN-MDSCs from HIV-1-infected patients have been shown to inhibit T cell proliferation *via* both PDL-1/PD-1 interaction and ROS release (Bowers et al, 2014), whereas those from septic patients have been reported to inhibit T cell proliferation *via* ARG-1 release (Darcy et al, 2014; Uhel et al, 2017), ROS (Janols et al, 2014) or PDL-1 (Charoensappakit et al, 2025), and those from pregnant women *via* ARG-1 and ROS (Kostlin et al, 2016) or PD-L2 (Li et al, 2020). Therefore, whether the inhibition of T cell proliferation and activation through mechanisms other than ARG-1 release reflects the existence of subpopulations of human PMN-MDSCs strictly associated with the underlying disease remains to be clarified.

## Mature PMN-MDSCs exert the immunosuppressive activities attributed to PMN-MDSCs

Mostly influenced by findings in mice (Grover et al, 2025), it has long been assumed that PMN-MDSCs consist of immature neutrophil precursors also in humans (Brandau et al, 2011; Gondois-Rey et al, 2021; Grover et al, 2025; Solito et al, 2011) and, therefore, have been investigated as a single-cell population, without considering that they actually consist of a heterogeneous mixture of **m**PMN-MDSCs and PRINs (in variable proportions from patient to patient) (Bergenfelz and Leandersson, 2020; Scapini et al, 2016). Once their mixed composition was ascertained, it became obvious to ask which one of the two cell fractions is effectively responsible for the immunosuppressive activities. To address this question, we took advantage of the remarkable abundance of circulating PMN-MDSCs in GDs (Luyckx et al, 2012; Vasconcelos et al, 2003) to sort them based on the expression of CD10 [a well-known marker of mature neutrophils (Elghetany, 2002)] and hence obtained CD10^-^PRINs and CD10^+^**m**PMN-MDSCs from the LDN fraction. By functionally evaluating them in comparison to HD NDNs, we could demonstrate that the **m**PMN-MDSCs from LDNs of GDs, but not the PRINs, display the most potent “immunosuppressive” activities toward T cells, *via* cell-to-cell contact-dependent ARG-1 release (Marini et al, 2017). The same findings were subsequently confirmed by other groups, who sorted both **m**PMN-MDSCs and PRINs from LDNs of patients with HNC (Lang et al, 2018), urological cancer (UC) (Lang et al, 2018), lung cancer (Vanhaver et al, 2024), or from total BM neutrophils of patients with multiple myeloma (MM) (Perez et al, 2020) and found that the **m**PMN-MDSC fraction exhibits a greater capacity to suppress T cell proliferation compared to the PRINs. CD10 can be instrumental in distinguishing **m**PMN-MDSCs from PRINs (Gondois-Rey et al, 2021; Marini et al, 2017; Mehmeti-Ajradini et al, 2020; Tavukcuoglu et al, 2020; Vanhaver et al, 2024); however, it is also important to note that CD10 should be considered a marker of mature neutrophils only, and not a PMN-MDSC-specific marker. Whether human **m**PMN-MDSCs constitute the most potent suppressive subset of PMN-MDSCs in diseases other than cancer remains to be systematically demonstrated. In this context, it is worth mentioning a study in which, in line with our findings on GDs, mature CD10^+^ and immature CD10^-^LDNs were isolated from patients with sepsis and tested for their ability to inhibit T cell activity (Liu et al, 2022). The study concluded that only CD10^+^LDNs exhibit immunosuppressive functions (Liu et al, 2022). However, most studies on PMN-MDSCs in diseases unrelated to cancer have primarily focused on total PMN-MDSCs, rather than isolating and directly comparing the immunosuppressive capacities of their mature and immature fractions.

Surprisingly, we also found that the CD10^+^**m**PMN-MDSCs of NDNs from GDs exhibit immunosuppressive activities toward T cells (via ARG-1 release) at identical levels to those of CD10^+^**m**PMN-MDSCs from LDNs (Marini et al, 2017), thus contradicting the general assumption that PMN-MDSCs exhibit altered buoyancy and exclusively settle within the mononuclear cell layer. Indeed, GDs are not the only subjects whose NDNs display immunosuppressive activities in addition to mLDNs, as is also the case for the NDNs of septic patients (Liu et al, 2022) and those with HIV (Bowers et al, 2014). It therefore appears that, under conditions characterized by severe systemic inflammation, the emergency granulopoiesis-mediated reprogramming becomes so accelerated that also the mNDNs, in addition to mLDNs, acquire the phenotypic and functional features of **m**PMN-MDSCs.

By contrast, **m**PMN-MDSCs of cancer patients are rarely recovered from NDNs because of their typical mobilization in low numbers (Bergenfelz and Leandersson, 2020; Scapini et al, 2016). Nonetheless, since systemic inflammation and emergency granulopoiesis might also exacerbate in cancer patients, especially at advanced stages (Aliazis et al, 2024; Manz and Boettcher, 2014), the fact that a few studies have reported the presence of **m**PMN-MDSCs within the NDNs is not surprising (Choi et al, 2012; Tsuda et al, 2012; Vanhaver et al, 2024). Although additional work is necessary to elucidate such issues, these observations highlight the importance of analyzing LDNs and NDNs separately across all clinical conditions where the presence of PMN-MDSCs is anticipated. They also indicate that using altered buoyancy as the sole criterion for differentiating NDNs from **m**PMN-MDSCs might be misleading. Accordingly, neutrophils that suppress T cell functions and exhibit phenotypic and transcriptional characteristics typical of PMN-MDSCs should be designated as such, regardless of their buoyancy. This would avoid the nomenclatural inconsistencies reported in studies of, for example, sepsis (Darcy et al, 2014; Janols et al, 2014; Kwok et al, 2023; Liu et al, 2022; Uhel et al, 2017), HIV infection (Bowers et al, 2014), MM (Lewinsky et al, 2021; Perez et al, 2020), and GDs (Lattanzi et al, 2025; Luyckx et al, 2012; Marini et al, 2017; Montaldo et al, 2022; Pettinella et al, 2024; Vasconcelos et al, 2003) in which neutrophils that suppress T cell functions are sometimes designated as PMN-MDSCs when isolated from the LDN fraction, whereas they are referred to more generally as immunosuppressive neutrophils when obtained from the NDN fraction, whole blood, or total  BM aspirates.

## Additional immunologic functions of PMN-MDSCs

While it is unknown whether in vitro-activated NDNs acquiring immunosuppressive properties can target immune cells other than T cells, PMN-MDSCs have been described to interact with NK cells (consequently inhibiting their cytotoxicity and cytokine production ability) (Pettinella et al, 2024; Rieber et al, 2013; Tumino et al, 2020; Tumino et al, 2021), and innate lymphoid cells type 2 (ILC2, consequently inhibiting their cytokine production ability) (Cao et al, 2019). In addition, PMN-MDSCs from patients with bladder cancer (Eruslanov et al, 2012) or HIV infection (Vollbrecht et al, 2012) have also been shown to promote the expansion of CD4⁺FoxP3⁺ regulatory T cells (Tregs), whereas PMN-MDSCs from pregnant women have been reported to induce the expansion of both Tregs and type 2 helper T (Th2) cells (Kostlin et al, 2016). These findings suggest that, in addition to inhibiting the functions of antitumor CD4⁺ Th1 and CD8⁺ cytotoxic T cells, PMN-MDSCs may also drive T cell polarization toward the expansion of pro-tumorigenic/anti-inflammatory T cell subsets. It is worth noting that not only are the molecular mechanisms underlying these effects poorly characterized, but also whether **m**PMN-MDSCs, PRINs, or both are responsible for the previously described effects remains unclear. For instance, some studies have demonstrated that total PMN-MDSCs from pregnant women (Rieber et al, 2013), cancer patients (Tumino et al, 2021), or GDs (Tumino et al, 2020), inhibit NK cell functions. On the other hand, we showed that **m**PMN-MDSCs, but not PRINs, are responsible for this inhibitory capacity [(Pettinella et al, 2024) and our unpublished observations].

We also have limited knowledge of whether **m**PMN-MDSCs and PRINs perform other effector functions of HD NDNs at normal levels, such as phagocytosis, respiratory burst activity, degranulation, chemotaxis, NETosis, and cytokine production. For instance, we found that **m**PMN-MDSCs from GDs exhibit reduced cytotoxicity toward K562 tumor cells (Pettinella et al, 2024). Other studies employing total PMN-MDSCs from patients with cancer (Brandau et al, 2011; Eruslanov et al, 2012; Saraiva et al, 2021), sepsis (Sun et al, 2022), or GDs (Montaldo et al, 2022), have reported inconsistent and, at times, contradictory results regarding their capacity to produce cytokines or ROS, to perform phagocytosis, or to exhibit chemotactic activity. It is likely that these controversial results are attributable to the highly variable ratio of mature and immature cells within the total PMN-MDSC populations, which is influenced by disease type and severity. It is therefore evident that the latter findings will become definitively informative once isolated **m**PMN-MDSCs and/or PRINs are directly compared with control neutrophils at equivalent maturation stages. Hence, once more elements about the functional, non-immunosuppressive capacities of PMN-MDSCs are accumulated, they might be utilized as additional criteria to distinguish and categorize **m**PMN-MDSCs/PRINs from the other types of “immunosuppressive” neutrophils. Finally, although the potential beneficial effects of the “immunosuppressive” actions by PMN-MDSCs are still poorly investigated in humans, they do exist. For instance, total PMN-MDSCs have been proposed to play a critical role in promoting maternal-fetal tolerance during pregnancy, thereby preventing miscarriage and supporting fetal development (Kostlin-Gille and Gille, 2020), as well as to help mitigate excessive immune responses in sepsis, trauma, and certain inflammatory and autoimmune diseases (Ostrand-Rosenberg et al, 2023). It is conceivable that PMN-MDSCs are part of a physiological regulatory network that, under chronic stimulation, promotes their accumulation to exert pathogenic effects.

## Transcriptomic features related to mature PMN-MDSCs

Whether PMN-MDSCs exhibit specific transcriptomic features associated with their immunosuppressive or other protumor functions has remained unexplored for a long time. To address this, some studies have reported data on the bulk RNA transcriptomes of PMN-MDSCs from cancer patients (Condamine et al, 2016) or GDs (Pelosi et al, 2021). However, once again, these studies did not consider that PMN-MDSCs consist of both mature and immature cells, which may possess distinct transcriptomic profiles specific to each cell type. Even the simple comparison between PMN-MDSCs and NDNs might be misleading, as it may reveal differences in gene expression that primarily reflect variations in maturation stages, rather than the inherent distinctiveness of these cells. Therefore, to comprehensively characterize the specific transcriptomic features of **m**PMN-MDSCs (the more potent immunosuppressive cells), we initially conducted bulk RNA sequencing of **m**PMN-MDSCs from patients with either HNC or non-small cell lung cancer (NSCLC) (precisely, using the CD10^+^cells from the LDN fractions only), as well as from GDs (in this latter case, using the CD10^+^cells from both the LDN and NDN fractions), to compare it with bulk RNA sequencing of NDNs from cancer patients and HDs (Pettinella et al, 2024). By such an approach, we ultimately identified a distinct gene signature of **m**PMN-MDSCs (from now on named “**m**PMN-MDSC gene signature”) that included 1930 genes associated not only with their immunosuppressive features, but also with several cancer-related biological processes (Box 2). This “**m**PMN-MDSC gene signature” was found enriched in the transcriptome of **m**PMN-MDSCs from the BM of MM patients, of total PMN-MDSCs from NSCLC patients, as well as associated with poor outcomes of patients affected by several types of cancers (Pettinella et al, 2024; Chang et al, 2025) Conversely, the **m**PMN-MDSCs signature was found absent in the transcriptome of either proinflammatory mature LDGs from SLE patients (as expected), or of mature immunosuppressive CD62L^low/dim^CD16^bright^ neutrophils isolated from voluntary HDs treated with very low doses of LPS (Pettinella et al, 2024). Consistently, CD62L^low/dim^ CD16^bright^ neutrophils have also been shown to inhibit T cell proliferation when freshly isolated from the blood of LPS-treated donors, but *via* the release of H₂O₂ and not of ARG-1 (Pillay et al, 2012), thereby indicating that they differ from **m**PMN-MDSCs. Notably, our “**m**PMN-MDSC gene signature” was also found to be enriched in a single-cell RNA-seq dataset (Kwok et al, 2023) of peripheral mature suppressive neutrophils from septic patients (C.L. et al, unpublished observations). Further research is therefore needed to define whether a common transcriptional reprogramming (likely induced by emergency granulopoiesis) across various disease contexts drives the differentiation into **m**PMN-MDSCs/PRINs.

Bioinformatic clustering analysis of our “**m**PMN-MDSC gene signature” then identified two major gene groups, namely g1 and g2 (see Box 2), whose inspection provided some fundamental information. In fact, the investigation into the expression of g1 genes in neutrophil precursors revealed that their transcripts are almost entirely downregulated in terminally differentiated NDNs from HDs, but not in **m**PMN-MDSCs of GDs and cancer patients, in which they are preserved at very high levels (Pettinella et al, 2024). An example is ARG-1, which, similarly to the other g1 genes, resulted in dramatic downregulation during neutrophil maturation into NDNs, but was maintained at significantly higher levels in **m**PMN-MDSCs (CL et al, unpublished observations). By contrast, g2 genes were found absent in HD immature neutrophils/NDNs; however, they were selectively upregulated in PRINs from GDs, starting from the metamyelocyte stage and persisting at high levels up to **m**PMN-MDSCs (Pettinella et al, 2024). Whether PRINs from cancer patients exhibit a similar pattern of g2 gene expression remains to be determined. Overall, although more data on the transcriptomic reprogramming of PRINs from cancer patients are needed to further validate this hypothesis, these findings suggest that PRINs and **m**PMN-MDSCs appear to represent different stages of maturation of the same cell type, which polarize towards an immunosuppressive (as well as protumor, see below) phenotype. In our view, PRINs and **m**PMN-MDSCs differentiation proceeds in parallel with the maturation of normal immature and mature neutrophils. These observations may also explain why some studies have reported that PRINs can exhibit immunosuppressive functions under specific conditions (Brandau et al, 2011; Gondois-Rey et al, 2021; Lang et al, 2018), although to a lesser extent than their mature counterparts (Lang et al, 2018).

Box 2 The mPMN-MDSC gene signature Recently, a distinct gene signature shared by **m**PMN-MDSCs from cancer patients and GDs was identified through a bulk RNA-seq experiment, and subsequently validated in TANs using single-cell RNA-seq experiments (Lattanzi et al, 2025; Pettinella et al, 2024). By overlapping such “**m**PMN-MDSC gene signature” with the transcriptomes of immature/mature neutrophils from either peripheral blood from GDs or BM aspirates from HDs, two major gene groups (g1 and g2) were identified by K-means clustering analysis (Pettinella et al, 2024). More specifically, among the g1 genes characterizing the related GO terms there were *ALDH2, ARG2*, and *SAT2* (associated to processes such as “arginine and proline metabolism”), *ACADM, HADHA*, and *CPT1A* (associated to “fatty acid and lipoprotein metabolism”), *ATP6V1F, COX7C, MT-CO3, MT-CO1, MT-CYB*, and *NDUFB7* [associated to “oxidative phosphorylation and energy (ATP) metabolism”], *PKM, ENO1, HK3*, and *LDHA* (associated to “glycolysis”), and *PSMB6, PSMB7, RBX1*, and *PSMA1* (associated to “response to oxidative stress”) (Pettinella et al, 2024). By contrast, biological processes such as “negative regulation of leukocyte-mediated immunity”, “negative regulation of cytokine production”, “negative regulation of leukocyte-mediated cytotoxicity” characterized the g2 group, and included genes such as *CD52*, *CD84*, *EP2, SMAD3, LGALS1*, and *CX3CR1* (Pettinella et al, 2024). Furthermore, the “**m**PMN-MDSC gene signature” was also found to include previously proposed PMN-MDSC associated-genes, such as *LIRB1*, *OLR1/LOX1*, and *SPARC*, or other genes typically associated with tumor progression, including those favoring angiogenesis, such as *MMP8*, and *MMP9*, or regulating the epithelial-mesenchymal transition (EMT), such as *VIM*, *TPM1*, *TPM4*, *CD44*, and *ITGB1* (Pettinella et al, 2024).

## The task to discover specific markers of human mPMN-MDSCs

A major cause that has hampered a more rapid/detailed characterization of human PMN-MDSCs in general, at the epidemiologic, molecular, and functional levels, is the lack of knowledge on potentially specific markers that could reliably distinguish PMN-MDSCs from normal mature and immature neutrophils, as well as from other types of immunosuppressive neutrophils or proinflammatory LDGs. In fact, human PMN-MDSCs have long been characterized by the CD66b^+^CD15^+^CD14^−/dim^CD33^dim^HLA-DR phenotype (Cassetta et al, 2019), hence representing a general neutrophil phenotype. Subsequently, they have also been shown to express chemokine receptors (e.g., CXCR2, CXCR4), activation (e.g., CD62L, CD54/ICAM-1, CD63, CD274/PD-L1) and functional markers (ARG-1) (Zea et al, 2005), Lectin-like oxidized low-density lipoprotein (LDL) receptor-1 (LOX-1) (Condamine et al, 2016) or CD36 (Al-Khami et al, 2017), at variable levels compared to NDNs, depending on the disease type and severity (Scapini et al, 2016). However, most of these markers have neither been validated across different disease models nor evaluated in terms of differential expression between mature and immature cells. In fact, all studies aimed at identifying surface markers of human PMN-MDSCs, similarly to those used to define their functions or gene signatures, have used total PMN-MDSCs, rather than separately analyzing **m**PMN-MDSCs and PRINs. For instance, the study by Condamine and coworkers (Condamine et al, 2016), which characterized a gene signature of total PMN-MDSCs from lung cancer patients, indicated LOX-1 (encoded by *ORL1*) as a specific marker for PMN-MDSCs. Since then, LOX-1 has become one of the most widely used markers for detecting PMN-MDSCs in patients with cancer (Chai et al, 2019; Kim et al, 2019; Nan et al, 2018) or various pathological conditions (Coudereau et al, 2022; Deng et al, 2024; Li et al, 2021; Rahman et al, 2019). However, according to our **m**PMN-MDSCs gene signature, *ORL1* (included in g1 genes) and, consequently, LOX-1 are already expressed at elevated levels in both PRINs and HD immature neutrophils (Pettinella et al, 2024). Consistent with these latter findings, and irrespective of their different immunoregulatory functions, not only CD66b^+^ PMN-MDSCs from patients with various cancer types (Chai et al, 2019; Condamine et al, 2016; Kim et al, 2019; Nan et al, 2018), trauma (Li et al, 2021) sepsis and severe COVID-19 (Coudereau et al, 2022), but also proinflammatory CD66b^+^ LDGs from patients with SLE (Rahman et al, 2019) or allergic rhinitis (Deng et al, 2024) or even CD66b^+^LDNs from HDs (St Laurent et al, 2025) have been found to display remarkable levels of LOX-1 expression, likely determined by the immature neutrophil fraction therein present. Nevertheless, although the utilization of LOX-1 as a marker for circulating PMN-MDSC appears contradictory, human immunosuppressive/protumor TANs have been found to express *ORL1* (by bulk or scRNA-seq experiments) (Lattanzi et al, 2025; Salcher et al, 2022; Veglia et al, 2021), as well as LOX-1 protein (by both flow cytometry and immunohistochemistry) (Salcher et al, 2022; Si et al, 2019). For example, a subpopulation of TANs in HNC expressing LOX-1 and ARG-1 was found to be physically associated with tumor-infiltrating lymphocytes and to reduce their antitumor functions (Si et al, 2019). These latter findings suggest that LOX-1 could eventually serve as a reliable and specific marker for protumor TANs, which, unlike circulating PMN-MDSCs, predominantly include mature neutrophils only (Kadota et al, 2015; Lattanzi et al, 2025; Rotondo et al, 2009; Si et al, 2019).

Novel insights into the identification of specific markers of **m**PMN-MDSCs have been provided by the g2 genes of our “**m**PMN-MDSC gene signature”. In fact, considering that g2 genes showed the strongest differential expression between HDs and **m**PMN-MDSCs, we focused on them to identify mRNAs encoding surface molecules that could be used as markers associated with **m**PMN-MDSCs. Accordingly, we identified CD84, PTGER2, and CD52 mRNAs (Pettinella et al, 2024). Consistently, high surface levels of CD52, CD84, and PTGER2 antigens were specifically shared by **m**PMN-MDSCs from GDs and patients with NSCLC, HNC, diffuse large B-cell lymphomas (DLBCL), RCC, and transitional cell carcinoma (TCC), while they were not detected, or detected at very low levels, on either mature or immature neutrophils from the BM of HDs. CD52, CD84, and PTGER2 were also found to be expressed on PRINs cancer patients and GDs, although at lower levels compared to their autologous **m**PMN-MDSCs (Pettinella et al, 2024), suggesting that the proteins encoded by these g2 genes are progressively upregulated during the maturation of PRINs to **m**PMN-MDSCs. It should be noted that CD84 expression was previously detected on Mo-MDSCs and total CD15^+^CD14^-^CD11b^+^HLA-DR^-^neutrophils (exerting immunosuppressive activities) from the BM of the MM patients (Lewinsky et al, 2021). Moreover, the capacity of PGE2 to induce MDSCs (Porta et al, 2020; Rodriguez-Ubreva et al, 2017), the role of soluble CD52 in mediating T cell immunosuppression (Bandala-Sanchez et al, 2018) and the function of CD84 as an upregulator of PDL-1 on MDSCs (Lewinsky et al, 2021) imply that these surface molecules may be involved in the discrete functions of these cells. However, whether CD52, CD84, and PTGER2 are co-expressed by a unique subpopulation of **m**PMN-MDSCs or whether they are sparsely expressed across all **m**PMN-MDSCs remains unknown. It also remains to be investigated whether these molecules are expressed by **m**PMN-MDSCs in pathological conditions beyond cancer.

Whatever the case may be, the recognition of specific, universally recognized markers will significantly advance the field, as it will facilitate the prompt identification/isolation/sorting of **m**PMN-MDSCs and PRINs, thereby circumventing the need for laborious and time-consuming functional assays. Based on our experience, to define specific PMN-MDSC markers/gene signatures, one must: (i) make sure to isolate pure populations of immunosuppressive *versus* non-immunosuppressive neutrophils at an identical maturation stage; (ii) focus on genes/markers that are not expressed (or expressed at negligible levels) either on mature or on immature neutrophils from HDs. We believe that identifying a combination of multiple specific markers expressed by **m**PMN-MDSCs provides greater accuracy than a single marker. The notion that **m**PMN-MDSCs or PRINs are specifically recognized by a combination of markers is more appropriate as it would not only better reflect the complex level of reprogramming that **m**PMN-MDSCs undergo, but would circumvent the high plasticity of neutrophils (cells that easily modulate the expression of individual markers in different contexts), even independently of the reprogramming into PMN-MDSCs.

## Human PMN-MDSCs: immunosuppressive and protumor functions

Whether human PMN-MDSCs, besides being immunosuppressive, also promote tumor growth in cancer patients has remained poorly explored until recently. To our knowledge, in fact, only a single study shows that PMN-MDSCs from breast cancer patients directly promote tumor growth in a mouse xenograft model (Mehmeti-Ajradini et al, 2020). Another unanswered question is whether protumor TANs (often defined as N2s) derive from local neutrophils or from circulating NDNs and/or correspond to PMN-MDSCs that have migrated into tumor tissue. Since murine PMN-MDSCs have been shown to directly promote angiogenesis, epithelial-to-mesenchymal transition (EMT), and pre-metastatic niche formation (Grover et al, 2025), many researchers (particularly those working with experimental mouse models) utilize the term PMN-MDSCs to interchangeably refer to both the peripheral and tumor-tissue neutrophil populations (Eruslanov et al, 2025; Grover et al, 2025). Such terminology, therefore, assumes that PMN-MDSCs and TANs may represent the same cell type (Eruslanov et al, 2025; Grover et al, 2025). In this context, three recent scRNA-seq studies—one conducted in a mouse model of pancreatic ductal adenocarcinoma (PDAC) (Ng et al, 2024), one conducted in a mouse brain tumor model (Ugolini et al, 2025) and the other in PDAC patients (Wang et al, 2023)—have proposed that neutrophils differentiate into protumor cells only within the TME, but not in the circulation, spleen, or BM. Such local maturation of newly arrived neutrophils into protumor TANs has been shown to induce hyperactivated glycolysis, immunosuppressive and protumor/proangiogenic transcriptomic features, and to be driven by the hypoxic niche (Ng et al, 2024; Ugolini et al, 2025; Wang et al, 2023). Very recently, hypoxia has also been shown to remodel neutrophils, conferring immunosuppressive functions by inducing EGR1 (Chang et al, 2025), a transcription factor whose mRNA is present in the **m**PMN-MDSC signature (Pettinella et al, 2024). Therefore, according to these findings (Ng et al, 2024; Ugolini et al, 2025; Wang et al, 2023), circulating and tumor-infiltrating neutrophils do not undergo a common reprogramming process, thereby excluding the possibility that PMN-MDSCs and TANs may share phenotypic and functional similarities or even represent the circulating and tissue counterparts of the same cell population. Since other studies performed in cancer patients reported similar conclusions (Xue et al, 2022; Zilionis et al, 2019), it is fundamental to emphasize that, in all of them, the bioinformatic analyses were conducted by integrating transcriptomes from neutrophils derived from distinct anatomical sites (e.g., peripheral blood, spleen, BM, and tumor tissue) into a single dataset. This approach inherently favors the identification of gene expression differences driven by the tissue of origin rather than the intrinsic biological transcriptomic features of the distinct cell populations.

**Fig. 2:**
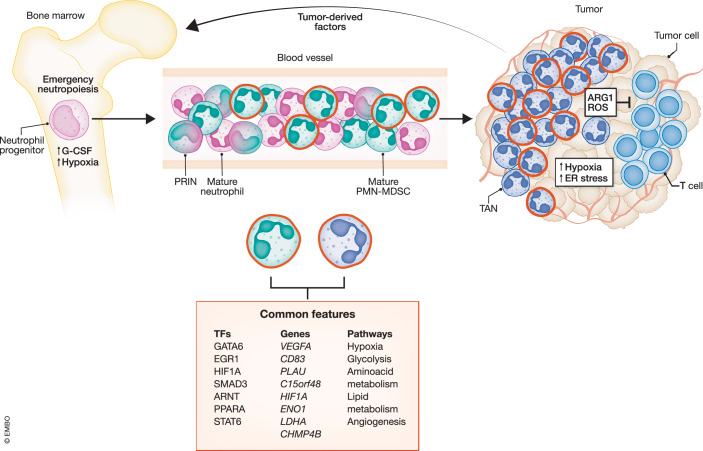
Tumor-driven emergency neutropoiesis drives the release into the circulation of aberrantly matured PRINs and **m**PMN-MDSCs sharing common transcriptomic features with TANs. In cancer patients, tumor-derived soluble factors trigger an emergency granulopoiesis, consequently leading to a dysregulated BM reprogramming of neutrophils, which ultimately results in the release of PRINs and **m**PMN-MDSCs into the circulation. scRNA-seq experiments have uncovered that about half of **m**PMN-MDSCs and TANs share several common genes and transcription factors (TFs) associated with biological processes such as “responses to hypoxia and oxidative stress”, “positive regulation of angiogenesis”, and “altered cell metabolism” (namely “glycolytic process”, “arginine and proline metabolic process”, “regulation of lipid metabolic process”).

To further investigate whether, in humans, there are transcriptomic similarities between **m**PMN-MDSCs and protumor TANs, we performed scRNA-seq experiments of NDNs and **m**PMN-MDSCs from patients with NSCLC (chosen as a reference cancer model), and **m**PMN-MDSCs from GDs to compare their single-cell transcriptomes to publicly available scRNA-seq TAN datasets from 14 different tumor types (Lattanzi et al, 2025; Salcher et al, 2022; Wu et al, 2024). Besides NSCLC, the latter datasets included colon adenocarcinoma, gallbladder cancer, hepatocellular carcinoma, renal cell carcinoma, oral squamous cell carcinoma, and pancreatic adenocarcinoma (Lattanzi et al, 2025; Salcher et al, 2022; Wu et al, 2024). Our additional aim was to verify whether the “**m**PMN-MDSC gene signature” is expressed by all **m**PMN-MDSCs or only by a fraction of them. However, unlike previous studies (Wang et al, 2023; Xue et al, 2022; Zilionis et al, 2019), we have initially compared each neutrophil population to its appropriate internal control (e.g., NSCLC **m**PMN-MDSCs *versus* autologous mNDNs and HD mNDNs; GD **m**PMN-MDSCs *versus* HD mNDNs; tumor TANs *versus* neutrophils infiltrating the normal adjacent tissue—NANs) to first identify specific NSCLC and GD **m**PMN-MDSC, as well as TAN, clusters, and then analyzed their specific transcriptomic similarities. This approach enabled us to detect more subtle transcriptomic features of the underrepresented **m**PMN-MDSC population. We, in fact, identified seven distinct scRNA-seq cell clusters in **m**PMN-MDSCs from NSCLC patients; one of these clusters (i.e., NSCLC c6 , representing ~50% of the total cells) was specifically enriched with immunosuppressive and protumor genes of the “**m**PMN-MDSC gene signature” (Lattanzi et al, 2025). Similarly, nine distinct pan-cancer TAN clusters, three of which (i*.*e., TAN c6-c8, representing approximately 50% of the total cells specifically enriched with protumor and immunosuppressive genes) were identified across the different, previously listed tumor types (Lattanzi et al, 2025). By comparing the DEGs between NSCLC c6 and TAN c6-c8, we subsequently identified 60 commonly expressed genes, including *VEGFA, IL1B, CD44, CD83, PLAUR, LDHA, PLAU, HIF1A*, *ENO1, PKM, LDHASP1, ATF2, XBP1, GATA6, TWIST1*, as well as 123 commonly active transcription factor regulons, most of them associated with biological processes, such as “responses to hypoxia and oxidative stress”, “positive regulation of angiogenesis”, and “altered cell metabolism” (namely “glycolytic process”, “pyruvate metabolic process”, “regulation of lipid metabolic process” and “lactate metabolic process”) (Lattanzi et al, 2025). Notably, eight distinct cell clusters were identified in **m**PMN-MDSCs from GDs, four of which (representing approximately 80% of the total cells) were significantly enriched in the 60 genes commonly expressed by NSCLC c6 and TAN c6-c8, and displayed activation of the hypoxia signaling pathway and metabolic reprogramming (Fig. 2). That **m**PMN-MDSCs exhibit metabolic alterations, including glycolysis, arginine and proline metabolism, and fatty acid oxidation, is already well-documented and thought to be closely linked to their immunosuppressive functions (Goldmann and Medina, 2024). Less expected was the activation of hypoxia signaling in **m**PMN-MDSCs, even under non-tumor conditions, such as the GDs. We suspect that activation of the hypoxia signaling might stem from emergency granulopoiesis shared by both cancer and GD individuals (Aliazis et al, 2024; Manz and Boettcher, 2014; Pedersen et al, 2016), which exposes BM progenitor cells to a hypoxic and tumor-like environment (Urao et al, 2022). PMN-MDSCs may consequently acquire a hypoxia-induced metabolic program during BM maturation, which may persist in circulation. Additionally, cytokines and tumor-derived factors can further activate HIF-1α in the circulation independently of oxygen, thereby reinforcing hypoxia signaling and metabolic reprogramming.

While further research is required to confirm these hypotheses, these data collectively provide evidence that human **m**PMN-MDSCs and TANs share scRNA-seq cell clusters that display common and distinct immunosuppressive/protumor transcriptional features. They also suggest a potential direct relationship between these two cell types, challenging the notion that they represent distinct entities. Although a more precise characterization of autologous **m**PMN-MDSCs and TANs, in terms of functional activities and phenotypical similarities, is needed, the identified similarities in their gene expression patterns indicate that human **m**PMN-MDSCs and TANs may be closely related, potentially representing different stages or phases of the same cellular entity, present in different anatomical locations.

## Conclusions

An important objective of cancer research on neutrophils is to delineate both the intrinsic and shared phenotypic, transcriptional, and functional features of human **m**PMN-MDSCs and TANs, whose current knowledge is summarized below and in Table 1. As already mentioned, PMN-MDSCs are generated in vivo as part of an emergency granulopoiesis process that alters the maturation and reprograms neutrophil precursors. Such emergency granulopoiesis-dependent differentiation and mobilization of PMN-MDSCs represent a key feature of these cells and, as such, constitute a criterion for distinguishing them from other types of immunosuppressive, emergency granulopoiesis-independent neutrophils. It should be noted, in this context, that the issue of considering PMN-MDSCs as a functional state of neutrophils rather than a distinct cell type has been raised (Quail et al, 2022). In our view, a “functional state” should more appropriately be referred to as a specialized activity acquired by fully mature neutrophils in response to physiological or pathological contexts, because of their intrinsic plasticity. For instance, we mentioned in this Perspective that mature neutrophils, when treated with appropriate cues, may acquire new functional states, thereby performing immunosuppressive activities. By contrast, since PMN-MDSCs arise from a complex reprogramming process, they should be regarded as cells that follow a differentiation trajectory associated with specific physiologic and pathological conditions and, as such, should retain their distinct terminology. It is plausible that PMN-MDSC reprogramming initiates in the BM, is maintained once the cells enter the bloodstream, and ultimately completes within the TME. The initial emergency granulopoiesis process leads to the development of PRINs (Table 1), which ultimately mature into circulating **m**PMN-MDSCs. As highlighted in this Perspective, **m**PMN-MDSCs exhibit distinct transcriptional and phenotypic features (Table 1) and, unlike other immunosuppressive neutrophils, induce a T cell anergic state primarily through ARG-1 release rather than T cell killing (Table 1). Novel insights are expected from a more comprehensive characterization of the molecular features of PRINs (Table 1), which will also help elucidate the mechanisms underlying the altered maturation that leads to the generation of **m**PMN-MDSCs, including the involvement of potential master transcriptional regulators and epigenetic modifications. Not to mention that further research is necessary to achieve a comprehensive characterization of the mediators and the molecular mechanisms responsible for the **m**PMN-MDSC reprogramming occurring during emergency granulopoiesis. The mere induction of an emergency granulopoiesis process, in fact, is not sufficient per se to promote the generation of PMN-MDSCs, because in some diseases associated with emergency granulopoiesis, such as SLE, the final output cells are proinflammatory LDGs rather than PMN-MDSCs. Therefore, the PMN-MDSC reprogramming must require a fine balance of specific growth factors and mediators that drive this process under specific disease conditions. Understanding these factors and underlying mechanisms would enable the expansion and in vitro production of these cells from neutrophil precursors, thereby facilitating research on them. In this context, it is also essential to further validate the transcriptomic, phenotypic, and functional similarities between autologous **m**PMN-MDSCs and TANs in cancer patients (Table 1). Such studies will be instrumental in advancing our understanding and, ultimately, in facilitating the development of novel therapeutic strategies that selectively target these pathological neutrophil populations in both blood and tissues across various tumor types.

**Table 1:**
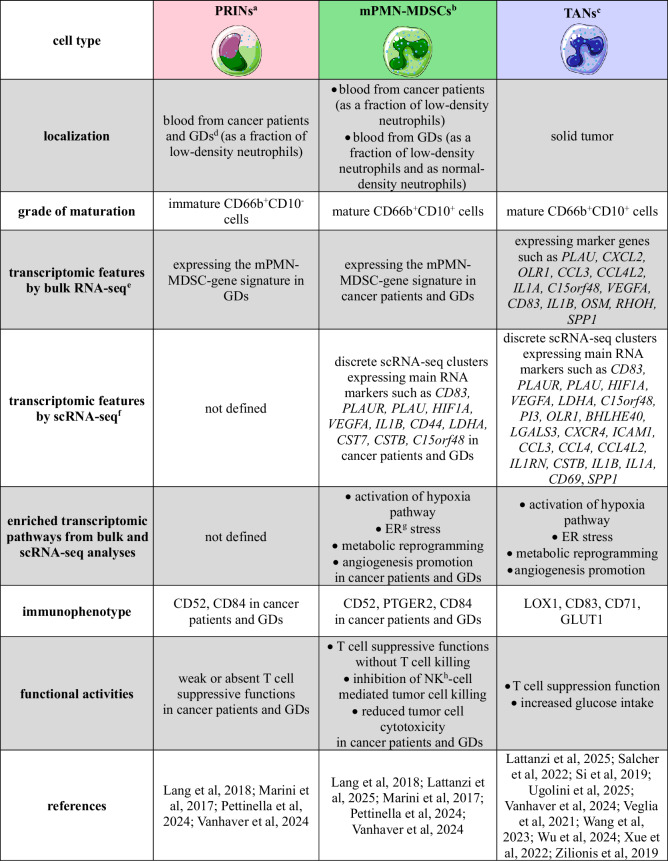
Main features of PRINs and mPMN-MDSCs (from cancer patients and GDs), as well as of TANs.

^a^PMN-MDSC-reprogrammed immature neutrophils;

^b^mature PMN-myeloid-derived suppressor cells;

^c^tumor-associated neutrophils;

^d^G-CSF-treated healthy donors;

^e^RNA-sequencing;

^f^single-cell RNA-seq;

^g^endoplasmic reticulum;

^h^natural killer.

In sum, human PMN-MDSCs appear to play a crucial role in both immunosuppression and tumor promotion and progression, on the one hand, but also in suppressing excessive immune responses and promoting immune tolerance, on the other hand. A better understanding of the biology of PMN-MDSCs and other immunosuppressive neutrophil populations will help determine an eventual, more appropriate nomenclature for these cells (Ng et al, 2025).

## Supplementary information

Peer Review File
