# Supplementary material for: Human immunosuppressive neutrophils: recent answers to old and new questions
Source: EMBO J. 2026 Jun 5;45(15):5193–206. doi: 10.1038/s44318-026-00829-6 (PMC13434422; doi:10.1038/s44318-026-00829-6)
Supplement: Supplementary file 1 [file 44318_2026_829_MOESM1_ESM.pdf]

THE EMBO JOURNAL

Human immunosuppressive neutrophils: recent answers to old and new questions

Patrizia Scapini, Chiara Lattanzi, Sven Brandau, and Marco Cassatella

Corresponding author(s): Marco Cassatella (marco.cassatella@univr.it) , Patrizia Scapini (patrizia.scapini@univr.it)

Review Timeline:

Submission Date:	6th Jan 26
Editorial Decision:	13th Feb 26
Revision Received:	28th Mar 26
Editorial Decision:	30th Apr 26
Revision Received:	5th May 26
Accepted:	22nd May 26

Editor: Ieva Gailite

Transaction Report:

(Note: With the exception of the correction of typographical or spelling errors that could be a source of ambiguity, letters and reports are not edited. Depending on transfer agreements, referee reports obtained elsewhere may or may not be included in this compilation. Referee reports are anonymous unless the Referee chooses to sign their reports.)

Dear Marco,

Thank you for submitting your Perspective article to The EMBO Journal. I sincerely apologise for the unusually protracted assessment process for your manuscript. I have now received input from two reviewers with expertise in neutrophil biology and diversity, which are attached below.

As you will see, both reviewers appreciate the topic and the timeliness of the article, and indicate that it should be published in our journal. At the same time, they present differing assessments of the article, with reviewer #1 asking for mainly minor textual edits, including a clearer delineation of the properties defining PMN-DSCs or other neutrophil subtypes (points 7 and 11). On the other hand, reviewer #2 approaches the article from a different perspective, indicating that they do not find the introduction of a strict and deterministic categorisation of the likely more context-dependent neutrophil specialisation as separate lineages helpful for the research field. Reviewer #2 encourages consideration of neutrophil subtypes as part of a continuum of variation in neutrophil production and function in response to the physiological and disease states. I strongly encourage you to take this input on board throughout the manuscript during the revision.

Taken together, based on the positive assessments of the reviwers regaridng the timeliness and relevance of the article, I would like to invite you to submit a revised version of the manuscript with reviewers' suggestions incorporated. From the editorial side, I would like to ask you to avoid the use of the abbreviation "PMN-MDSCs" in the article title, as it will likely reduce its accessibility to our broader readership.

There are also a few formatting requests that we would like to ask you to incorporate in the final version:
1. Please remove figures and the table from the manuscript text file, while leaving the figure legends in.
2. We are missing the ORCID iD for the co-corresponding author Patrizia Scapini. In order to link the ORCID iD to the account in our manuscript tracking system, the author in question has to do the following:
- Click the 'Modify Profile' link at the bottom of your homepage in our system.
- On the next page you will see a box halfway down the page titled ORCID*. Below this box is red text reading 'To Register/Link to ORCID, click here'. Please follow that link: you will be taken to ORCID where you can log in to your account (or create an account if you don't have one)
- You will then be asked to authorise Wiley to access your ORCID information. Once you have approved the linking, you will be brought back to our manuscript system.
Unfortunately, we cannot do this linking on the author's behalf for security reasons.
3. The Perspective format at our journal usually mandates inclusion of up to 50 references (https://link.springer.com/journal/44318/submission-guidelines#cms-Article-types). While I appreciate the scholarly nature of the reference list, please consider whetehr there are any non-essential references that could be removed without negatively affecting correct attribution of scientific contributions to the topic.
4. Please update references according to The EMBO Journal style. The articles should be listed alphabetically. Where there are more than 10 authors on a paper, the first 10 should be listed, followed by 'et al.'

I have also included in the attachment further details on figure preparation for the final version.

Please let me know if you have any questions about these points. I would be happy to discuss the revision further, also via a Zoom call should it be helpful.

Thank you for giving us a chance to consider your manuscript, and I look forward to receiving the revised version!

With best wishes,

Ieva

Ieva Gailite, PhD
Senior Scientific Editor
The EMBO Journal
Meyerhofstrasse 1
D-69117 Heidelberg
Tel: +4962218891309
i.gailite@embojournal.org

Instructions for preparing your revised manuscript:

Read our guidance for manuscript revisions and related editorial policies: https://link.springer.com/journal/44318/submission-guidelines#cms-Revised-submissions

Please make sure you upload a letter of response to the referees' comments together with the revised manuscript.

Please also check that the title and abstract of the manuscript are brief, yet explicit, even to non-specialists.

We realize that it is difficult to revise to a specific deadline. In the interest of protecting the conceptual advance provided by the work, we recommend a revision within 3 months (14th May 2026). Please discuss the revision progress ahead of this time with the editor if you require more time to complete the revisions. Use the link below to submit your revision:

https://emboj.msubmit.net/cgi-bin/main.plex

Referee #1:

The perspective piece by Scapini et al entitled "Human PMN-MDSCs: novel insights for old unanswered and newly emerging questions" addresses some confusion in the field of neutrophil MDSCs and provides a scan of the current evidence describing this subset. Particularly, which is appreciated, the authors are clear to state the discrepancies between mice and human MDSC data, which have contributed to some of the misunderstanding of their function and phenotype. The authors are well known in the field have contributed significantly to our understanding of PMN-MDSC, and this perspective will be highly regarded in the field. Overall, the text flows in a logical fashion, and touches the key points of this topic. However, the issues are presented in quite incoherently. It's unclear if PMN-MDSCs are immunosuppressive neutrophils as they share similar gene signatures with TANs as described. If they are distinct, how are they distinguished? The manuscript can be improved by clarifying certain arguments and other minor issues I list below in no order of importance:

1. In the summary, authors write a extremely long sentence beginning with "Nonetheless, usage of ..". This can be improved without the need for more words.
2. scRNA experiments should be "single cell transcriptomics" unless scRNA-seq is to be defined here.
3. The summary should give the readers a sense of what to expect from this perspective, currently it is unclear what the focus is. Notably, the ending sentence seems abrupt and unfinished.
4. On page 3, why is article italicized? This was not done on page 15.
5. The section "On the origin of human PMN-MDSCs" seems unhelpful and confusing to the main message of the perspective. Since the authors have stated that the origin is assumed based on mice, and that human PMN-MDSCs are different in mice, there is much speculation that does not add to the article. Authors propose to use their identified NCP populations to study this better but does not explain how they are superior to using subsequent precursors already characterized.
6. On page 4, the sentence beginning with "in our opinion..." is very long and hard to understand. The phrase in our opinion should be removed as it is understood as this sentence did not have a citation linked to it.
7. On page 5, the authors wrote "Naming any type of suppressive neutrophils as PMN-MDSCs is, in general, improper...." It would be beneficial to come out with a criteria for readers to consider in deciding if a neutrophil is a MDSC. Features like surface markers, functional tests performed, and gene signatures can be indicated. This could be a table or figure.
8. Page 6, the sentence beginning with "it should be mentioned..." should include direct references to the studies that demonstrate these functions and not cite references. In general, many reviews were used instead of the actual studies that support the arguments of this perspective.
9. Page 7 section goes against the cited reference 45, its confusing and is absent in the section.
10. On page 9, the text "...to display somewhat contradictory results..." is unclear and confusing what exactly is contradicting. Please clarify
11. On page 9, the text "Potentially, some of these functions might even counteract the "immunosuppressive" facet of PMN-MDSCs" is confusing and suggests that the term PMN-MDSCs can be misleading and inaccurate, and a more comprehensive nomenclature is preferrable. I suggest some way to help readers summarize what makes a neutrophil a TAN, a mPMN-MDSC, a NDN and a PRIN.
12. On page 13, the section describes the 3 PDAC studies explaining how protumoral neutrophils are not programmed in the BM or spleen. Does this indicate that PMN-MDSCs are distinct in that manner?
13. I am not sure if Fig 2B adds anything to the perspective. The GO processes are very general, and exploratory. What exact GO pathways is attributing to the PRINs or the mPMN-MDSC? For example, response to organic substances is vague to describe mPMN-MDSCs.
14. In figure 3, the word pathway in "hypoxia pathway" seems redundant. The title does not need to include "graphical abstract". The figure illustrates how similar TANs and mPMN-MDSCs are, which does not resolve the current misconception in the field. This also contradicts the PDAC studies cited, where blood and BM neutrophils are distinct from the TANs in the tumors. Hypoxia, for example, is typically upregulated in the TME as a response mechanism.
15. The R in PRINS indicate reprogramming, which is unclear why it was named as such. Could it be an activation, stimulated by the cytokine mileu. Reprogramming suggests something that has been changed upstream, which is not clear as of now (in

humans, as the authors write).

Referee #2:

This review article has the potential to be insightful; however, I have significant concerns regarding the terminology used, particularly the attempt to frame the complexity of neutrophil biology within the limiting concept of PMN-MDSCs. A central issue is that this term does not define a distinct cell type, but rather a functional state. As presented in this review, PMN-MDSCs represent a highly heterogeneous population comprising both immature and mature cells, which arise in contexts of systemic inflammation. There are many issues with this, which I will elaborate more in details below.

Firstly, while these contexts may indeed involve a broader shift toward immunosuppression, and presence of neutrophils at low density layers, there is currently no evidence that all cells within this heterogeneous neutrophil pool are immunosuppressive in vivo. Ex vivo assays can demonstrate global immunosuppressive potential, but they do not constitute evidence that such suppression occurs under physiological conditions. Consequently, it remains possible that a substantial fraction of cells encompassed by this definition fulfil functions unrelated to immunosuppression.

Most importantly, there is no evidence that PMN-MDSCs constitute a distinct cellular lineage. As also correctly discussed in this opinion article, these cells are most likely generated through emergency neutrophil production in response to inflammatory insults and therefore represent components of neutrophil regulatory circuits rather than a separate entity. To study these regulatory processes, especially as our understanding of this remains limited, it is essential to consider the underlying cell lineage when defining functional subsets, which here is simply neutrophils.

Therefore, while I understand the clinical need for a definition that captures potentially pathological neutrophil subsets in circulation, I question the benefit of continuing to use the term PMN-MDSCs. In my view, this terminology risks imposing an overly narrow conceptual framework on the study of neutrophil heterogeneity, function, and regulation. This concern is not unique to my perspective; several neutrophil experts have similarly argued against the continued use of this terminology already few years ago (10.1084/jem.20220011).

There are many important unresolved questions regarding neutrophil heterogeneity and its relationship to functions. Many of these stem from the fact that, in response to insults such as infections, different cancers, or tissue injuries, neutrophils engage multiple layers of regulation. These include the mode production (steady state versus emergency granulopoiesis) as well as the site of production (bone marrow versus extramedullary hematopoiesis), their persistence in the circulation (which influences their aging states) and the specialized activities they perform at sites of tissue damage/inflammation, from which they may potentially recirculate.
Given this, the key question in the field is not the one stated in this article "how PMN-MDSCs relate to a multitude of other neutrophil populations with immunosuppressive activity", but rather "how neutrophils are regulated at different levels to generate context-dependent and distinct functional pools".
From a clinical perspective, the relevant question is whether we can identify specific neutrophil subsets associated with particular disease contexts that are potentially pathogenic, informative of disease trajectory, or amenable to therapeutic targeting. However, to understand this, the conceptional framework must relate to the understanding the complexity of neutrophil regulation. Indeed, neutrophil heterogeneity is not inherently pathological: distinct neutrophil states are likely initially generated for specific purposes and in response to specific challenges. However, persistent stimulation due to unresolved inflammation may drive maladaptive responses and promote the sustained production of neutrophil populations with features that may indeed become pathogenic.
This is not a semantic debate. Terminology shapes how we conceptualize biological problems and directly influence both data interpretation and the design of future studies. Framing this biology through the lens of neutrophil regulation naturally encourages investigation into how neutrophil responses are shaped, and at what point regulation may become dysregulated. In contrast, a framework that promotes the idea that "alternative" neutrophils arise as a separate entity risks artificially separating these cells from normal neutrophil regulatory pathways and may ultimately limit our understanding of their development and ontogeny.

My advice is to reframe the concepts describe here without using the unhelpful and misleading terminology of PMN-MDSCs, this will also help stop propagating it further, so that bigger efforts in the field can concentrate better on the study of the functional heterogenicity of neutrophils and their biology.
Certainly, the very same concepts described in this review could be discussed equally well by shifting the way the authors think about (and describe) neutrophil regulation and diversity in circulation. The central theme of this article can be reframed around the question of how different immunosuppressive neutrophil populations relate to one another in circulation, at steady state and in response to challenge rather than how PMN-MDSCs relate to other immunosuppressive neutrophils.

UNIVERSITÀ di VERONA
Dipartimento di MEDICINA
DIRETTORE
Prof. Domenico Girelli

Point-by-point responses to the Reviewers

Reviewer #1:
The perspective piece by Scapini et al entitled "Human PMN-MDSCs: novel insights for old unanswered and newly emerging questions" addresses some confusion in the field of neutrophil MDSCs and provides a scan of the current evidence describing this subset. Particularly, which is appreciated, the authors are clear to state the discrepancies between mice and human MDSC data, which have contributed to some of the misunderstanding of their function and phenotype. The authors are well known in the field have contributed significantly to our understanding of PMN-MDSC, and this perspective will be highly regarded in the field. Overall, the text flows in a logical fashion, and touches the key points of this topic. However, the issues are presented in quite incoherently. It's unclear if PMN-MDSCs are immunosuppressive neutrophils, as they share similar gene signatures with TANs as described. If they are distinct, how are they distinguished?

We thank the Reviewer for the thorough evaluation of our manuscript and for her/his constructive criticisms, which we have substantially addressed.

The answer to the specific Reviewer's request: "*It's unclear if PMN-MDSCs are immunosuppressive neutrophils, as they share similar gene signatures with TANs as described*" is that, as clearly stated throughout the manuscripts, the term PMN-MDSCs refers to the circulating immunosuppressive neutrophils present in patients. The term PMN-MDSCs derives from the initial discovery of their intrinsic immunosuppressive properties. However, our recent findings demonstrate that human mPMN-MDSCs exhibit transcriptomic features beyond immunosuppression (PMID:38242120; PMID:40590753), some of which are shared with immunosuppressive/protumor TANs (PMID: 4059075). Therefore, as speculated at lines 508 - 512, mPMN-MDSCs and TANs in humans appear closely related, in our opinion, representing different maturational stages of the same cellular entity present in different anatomical locations.

The manuscript can be improved by clarifying certain arguments and other minor issues I list below in no order of importance:
1. *In the summary, authors write an extremely long sentence beginning with "Nonetheless, usage of...". This can be improved without the need for more words.*

The sentence has been rephrased and split into two sentences.

2. *scRNA experiments should be "single cell transcriptomics" unless scRNA-seq is to be defined here.*

We have defined single-cell RNA sequencing (scRNA-seq) experiments in the abstract and in the main text.

3. *The summary should give the readers a sense of what to expect from this perspective, currently it is unclear what the focus is. Notably, the ending sentence seems abrupt and unfinished.*

The abstract has been substantially rephrased, and the Perspective's focus is now more clearly emphasized in the final sentence.

4. *On page 3, why is article italicized? This was not done on page 15.*

We thank the Reviewer for bringing this to our attention. We have removed the term "article" since not necessary (its italicization was a typo mistake) and retained only the word "Perspective" throughout the manuscript.

5. *The section "On the origin of human PMN-MDSCs" seems unhelpful and confusing to the main message of the perspective. Since the authors have stated that the origin is assumed based on mice, and that human PMN-MDSCs are different in mice, there is much speculation that does not add to the article. Authors propose to use their identified NCP populations to study this better but does not explain how they are superior to using subsequent precursors already characterized.*

With all due respect, we disagree with the Reviewer's comment. Although the origin of human PMN-MDSCs has not been conclusively established, their heterogeneous composition - comprising both mature and immature cells - is considered indicative of a mobilization during emergency granulopoiesis. Such emergency granulopoiesis-dependent differentiation and mobilization of PMN-MDSCs constitute an important concept that warrants emphasis, as they distinguish PMN-MDSCs from other types of immunosuppressive neutrophils (as discussed in the "On the origin of human PMN-MDSCs" section and throughout the Perspective). In any case, we have rephrased and shortened lines 105 - 107 to improve clarity, as the Reviewer pointed out.
We also prefer to maintain the discussion of previous studies that attempted to generate PMN-MDSCs in vitro from neutrophil precursors. In fact, without a critical analysis of the studies published to date, one would conclude that the in vitro generation of human PMN-MDSCs has already been achieved, which, as outlined in lines 118 - 124, is not true.
Concerning NCPs, these cells constitute the earliest CD34+neutrophil-committed progenitors identified to date (PMID: 39939817; PMID: 35484408). As such, they demonstrate a substantially greater proliferative and expansion capacity than any downstream $CD34^-$neutrophil precursors, while retaining the ability to exclusively generate neutrophils. Such NCP properties render them particularly attractive for their ex vivo manipulation and reprogramming toward a PMN-MDSC phenotype. These concepts on NCPs are better explained in the revised manuscript, as requested by the Reviewer (lines 124 - 131).

6. On page 4, the sentence beginning with "in our opinion..." is very long and hard to understand. The phrase in our opinion should be removed as it is understood as this sentence did not have a citation linked to it.

The sentence mentioned by the Reviewer has been removed from the paragraph "On the origin of human PMN-MDSCs", rephrased and placed in the "Conclusions" section (lines –519 - 522).

7. *On page 5, the authors wrote "Naming any type of suppressive neutrophils as PMN-MDSCs is, in general, improper...." It would be beneficial to come out with a criteria for readers to consider in deciding if a neutrophil is a MDSC. Features like surface markers, functional tests performed, and gene signatures can be indicated. This could be a table or figure.*

To address the Reviewer's request, we have more clearly specified the criteria to define a neutrophil population as PMN-MDSC in the "Conclusions" section (lines 515 - 537), as well as in a Table (Table 1) newly added to the revised manuscript.

8. *Page 6, the sentence beginning with "it should be mentioned..." should include direct references to the studies that demonstrate these functions and not cite references. In general, many reviews were used instead of the actual studies that support this perspective's arguments.*

For the Reviewer's info, the sentence beginning with "it should be mentioned" has been shortened and rephrased in the revised manuscript (lines 180 - 181). The key references relevant to this section are listed throughout the paragraph.

Concerning the Reviewer's comment: "*in general, many reviews were used instead of the actual studies that support this perspective's arguments",* we would like to point out that, in the Perspective, we have always quoted original studies to support our arguments. It is, however, true that we have referred to review articles when the literature on a particular issue is too extensive to be cited comprehensively. Not to mention the necessity to comply with the Journal Editor's comment, which encouraged us to reduce rather than increase the number of cited references throughout the Perspective: "*The Perspective format at our journal usually mandates inclusion of up to 50 references (https://link.springer.com/journal/44318/submission-guidelines#cms-Article-types). While I appreciate the scholarly nature of the reference list, please consider whether there are any non-essential references that could be removed without negatively affecting correct attribution of scientific contributions to the topic."*

9. Page 7 section goes against the cited reference 45, its confusing and is absent in the section.

Assuming to have understood the Reviewer's concern, Reference 45 (40 in the revised manuscript) reports that promyelocytes display immunosuppressive properties, thereby supporting the dogmatic hypothesis that human PMN-MDSCs exhibit an immature phenotype, as observed in mice. However, as explained in the section "**Mature PMN-MDSCs exert the immunosuppressive**

activities attributed to PMN-MDSCs", several groups, including ours, have more convincingly demonstrated that the mature PMN-MDSCs exhibit the most potent immunosuppressive activity among total PMN-MDSCs. That, in humans, mature PMN-MDSCs are the most effective immunosuppressive cells among total PMN-MDSCs is adequately discussed, since it represents a fundamental advancement in the field, and a notion to always keep in mind.

10. On page 9, the text "...to display somewhat contradictory results..." is unclear and confusing what exactly is contradicting. Please clarify

We thank the Reviewer for highlighting the lack of clarity in the sentence that she/he mentions. We simply wanted to state that the results of published studies evaluating the functional capacity of PMN-MDSCs are inconsistent and, at times, contradictory. For instance, they report either increased or decreased cytokine or ROS production, as well as enhanced or impaired phagocytic and/or chemotactic activities by PMN-MDSCs. A plausible hypothesis is that the conflicting results arise from the highly variable composition of total PMN-MDSC populations, which are heterogeneous mixtures of mature and immature cells whose ratios are influenced by disease type and severity. The sentence/paragraph has been more clearly rephrased in the revised manuscript (lines 281 - 286)

11. On page 9, the text "Potentially, some of these functions might even counteract the 'immunosuppressive' facet of PMN-MDSCs" is confusing and suggests that the term PMN-MDSCs can be misleading and inaccurate, and a more comprehensive nomenclature is preferable. I suggest some way to help readers summarize what makes a neutrophil a TAN, a mPMN-MDSC, a NDN and a PRIN.

We have removed the sentence the Reviewer pointed out. As already mentioned in the reply to issue#7, we have summarized the specific features of PMN-MDSCs, PRINs, and TANs in a new Table (Table 1) included in the revised manuscript (see the "Conclusions" section of the Perspective; lines 515 - 537), as requested by the Reviewer.

12. On page 13, the section describes the 3 PDAC studies explaining how protumoral neutrophils are not programmed in the BM or spleen. Does this indicate that PMN-MDSCs are distinct in that manner?

As discussed in this Perspective (lines 455 - 461), the three PDAC studies mentioned in our Perspective have concluded that protumoral neutrophils exhibit noticeable transcriptomic reprogramming only after infiltrating the tumor tissue. This conclusion was reached using a bioinformatic approach that integrated neutrophil transcriptomes from distinct anatomical sites (e.g., peripheral blood, spleen, BM, and tumor tissue) into a single dataset. Notably, this approach inherently favors the identification of gene expression differences driven by tissue of origin rather than the intrinsic biological transcriptomic features of the distinct cell populations, and/or their eventual shared transcriptomic similarities. Consequently, an eventual reprogramming of

Dipartimento di Medicina
Sezione di Patologia Generale
Strada Le Grazie, 8 - 37134 Verona | T: +39 045 8027120
P. IVA 01541040232 | C.F. 93009870234

protumoral neutrophils in the BM, spleen, and circulation, before their infiltration into tumor tissue, was likely underestimated by the bioinformatic approach used in these studies. By contrast, we used a bioinformatic approach better suited to detect transcriptomic features of poorly represented cell populations (such as circulating mPMN-MDSCs) and, by doing so, we identified similarities between mPMN-MDSCs and protumoral/immunosuppressive TANs (lines 470 - 477). Therefore, as discussed in this Perspective (lines 508 - 512), and outlined above, human mPMN-MDSCs and TANs appear closely related, in our opinion, representing different maturational stages of the same cellular entity present in different anatomical locations. Obviously, further research is required to better clarify this issue.

13. I am not sure if Fig 2B adds anything to the perspective. The GO processes are very general, and exploratory. What exact GO pathways is attributing to the PRINs or the mPMN-MDSC? For example, response to organic substances is vague to describe mPMN-MDSCs.

We valued the Reviewer's perplexity about Figure 2B and removed it from the revised Perspective.

14. *In figure 3, the word pathway in "hypoxia pathway" seems redundant. The title does not need to include "graphical abstract". The figure illustrates how similar TANs and mPMN-MDSCs are, which does not resolve the current misconception in the field. This also contradicts the PDAC studies cited, where blood and BM neutrophils are distinct from the TANs in the tumors. Hypoxia, for example, is typically upregulated in the TME as a response mechanism.*

We have: 1) removed the words "graphical abstract" from the title of Figure 3 (Figure 2 in the revised version of the Perspective); 2) deleted the term "pathway" as correctly indicated by the Reviewer.

Concerning the Reviewer's statement: "*This also contradicts the PDAC studies cited, where blood and BM neutrophils are distinct from the TANs in the tumors*", our position is explained in the reply to comment #12. Regarding the Reviewer's statement: "Hypoxia, for example, is typically upregulated in the TME as a response mechanism", our bioinformatic approach identified not only transcriptomic features but also transcription factor regulons shared by both mPMN-MDSC and TANs, including the activation of the hypoxia pathway. As mentioned in our Perspective (lines 497 - 503), we hypothesize that activation of the hypoxia signaling in circulating mPMN-MDSCs stems from emergency granulopoiesis (shared by both cancer patients and GDs), which exposes progenitor cells to a hypoxic and tumor-like environment in the BM. PMN-MDSCs may consequently acquire a hypoxia-induced metabolic program during BM maturation, which may persist in circulation. Additionally, cytokines and tumor-derived factors can further activate HIF-1a in the circulation, independent of oxygen deprivation, thereby reinforcing hypoxia signaling and metabolic reprogramming.

15. The R in PRINS indicate reprogramming, which is unclear why it was named as such. Could it be an activation, stimulated by the cytokine mileu. Reprogramming suggests something that has been changed upstream, which is not clear as of now (in humans, as the authors write).

Our definition of immature PMN-MDSCs as PRINs (PMN-MDSC reprogrammed immature neutrophils) was intended to highlight their reprogramming toward mature PMN-MDSCs, as correctly noted by the Reviewer, and is supported by experimental evidence. First, as observed in our recent CRM paper (PMID: 38242120) and discussed in this Perspective (lines 341 - 343), PRINs from GDs express the g2 of the mPMN-MDSC gene signature, unlike immature neutrophils from HDs. Second, PRINs from both GDs and cancer patients, but not immature neutrophils from HDs, may express CD52 and CD84 (i.e., mPMN-MDSC markers), albeit not to the same extent as mPMN-MDSCs (lines 402 - 406). In our view, PRINs should be considered as the direct reprogrammed precursors of mPMN-MDSCs, rather than classical immature neutrophils (lines 532 - 534).

Dipartimento di Medicina
Sezione di Patologia Generale
Strada Le Grazie, 8 - 37134 Verona | T: +39 045 8027120
P. IVA 01541040232 | C.F. 93009870234

Reviewer #2:

This review article has the potential to be insightful; however, I have significant concerns regarding the terminology used, particularly the attempt to frame the complexity of neutrophil biology within the limiting concept of PMN-MDSCs. A central issue is that this term does not define a distinct cell type, but rather a functional state. As presented in this review, PMN-MDSCs represent a highly heterogeneous population comprising both immature and mature cells, which arise in contexts of systemic inflammation. There are many issues with this, which I will elaborate more in details below.

Firstly, while these contexts may indeed involve a broader shift toward immunosuppression, and presence of neutrophils at low density layers, there is currently no evidence that all cells within this heterogeneous neutrophil pool are immunosuppressive in vivo. Ex vivo assays can demonstrate global immunosuppressive potential, but they do not constitute evidence that such suppression occurs under physiological conditions. Consequently, it remains possible that a substantial fraction of cells encompassed by this definition fulfil functions unrelated to immunosuppression.

Most importantly, there is no evidence that PMN-MDSCs constitute a distinct cellular lineage. As also correctly discussed in this opinion article, these cells are most likely generated through emergency neutrophil production in response to inflammatory insults and therefore represent components of neutrophil regulatory circuits rather than a separate entity. To study these regulatory processes, especially as our understanding of this remains limited, it is essential to consider the underlying cell lineage when defining functional subsets, which here is simply neutrophils.

Therefore, while I understand the clinical need for a definition that captures potentially pathological neutrophil subsets in circulation, I question the benefit of continuing to use the term PMN-MDSCs. In my view, this terminology risks imposing an overly narrow conceptual framework on the study of neutrophil heterogeneity, function, and regulation. This concern is not unique to my perspective; several neutrophil experts have similarly argued against the continued use of this terminology already few years ago (10.1084/jem.20220011).

There are many important unresolved questions regarding neutrophil heterogeneity and its relationship to functions. Many of these stem from the fact that, in response to insults such as infections, different cancers, or tissue injuries, neutrophils engage multiple layers of regulation. These include the mode production (steady state versus emergency granulopoiesis) as well as the site of production (bone marrow versus extramedullary hematopoiesis), their persistence in the circulation (which influences their aging states) and the specialized activities they perform at sites of tissue damage/inflammation, from which they may potentially recirculate.

Given this, the key question in the field is not the one stated in this article "how PMN-MDSCs relate to a multitude of other neutrophil populations with immunosuppressive activity", but rather "how neutrophils are regulated at different levels to generate context-dependent and distinct functional pools".

From a clinical perspective, the relevant question is whether we can identify specific neutrophil subsets associated with particular disease contexts that are potentially pathogenic, informative of disease trajectory, or amenable to therapeutic targeting. However, to understand this, the conceptional framework must relate to the understanding the complexity of neutrophil

regulation. Indeed, neutrophil heterogeneity is not inherently pathological: distinct neutrophil states are likely initially generated for specific purposes and in response to specific challenges. However, persistent stimulation due to unresolved inflammation may drive maladaptive responses and promote the sustained production of neutrophil populations with features that may indeed become pathogenic.
This is not a semantic debate. Terminology shapes how we conceptualize biological problems and directly influence both data interpretation and the design of future studies. Framing this biology through the lens of neutrophil regulation naturally encourages investigation into how neutrophil responses are shaped, and at what point regulation may become dysregulated. In contrast, a framework that promotes the idea that "alternative" neutrophils arise as a separate entity risks artificially separating these cells from normal neutrophil regulatory pathways and may ultimately limit our understanding of their development and ontogeny.
My advice is to reframe the concepts describe here without using the unhelpful and misleading terminology of PMN-MDSCs, this will also help stop propagating it further, so that bigger efforts in the field can concentrate better on the study of the functional heterogenicity of neutrophils and their biology.
Certainly, the very same concepts described in this review could be discussed equally well by shifting the way the authors think about (and describe) neutrophil regulation and diversity in circulation. The central theme of this article can be reframed around the question of how different immunosuppressive neutrophil populations relate to one another in circulation, at steady state and in response to challenge rather than how PMN-MDSCs relate to other immunosuppressive neutrophils.

We thank the Reviewer for sharing her/his vision on the complexity of neutrophil biology. We acknowledge all the considerations raised by the Reviewer, which largely reflect the conceptual framework outlined in the PMID:35522219 article. We obviously agree with the Reviewer on the concept of neutrophil heterogeneity, a topic of great interest to us, which we have indeed addressed in some of our previous publications (e.g., PMID: 27558327; PMID: 31160207). Similarly to the Reviewer’s view, we also think that PMN-MDSCs are likely part of a physiological regulatory network - given their role in promoting maternal-fetal tolerance and in mitigating excessive immune responses - that, under chronic stimulation, promotes their accumulation to exert pathogenic effects (a concept that is now better specified in lines 297 - 299 of the revised Perspective).

On the other hand, we respectfully disagree with the Reviewer’s view that the PMN-MDSC term does not define “*a distinct cell type, but rather a functional state*”. In our view, a “functional state” more appropriately refers to a specialized activity acquired by fully mature neutrophils, reflecting their intrinsic plasticity in specific physiological or pathological contexts. In fact, as also mentioned in our Perspective, mature neutrophils may acquire specific “functional states” and perform, for instance, immunosuppressive activities (lines: 134; 526 - 532). By contrast, as explained in our Perspective (lines 528 -1532), PMN-MDSCs arise from a complex reprogramming process during neutrophil development that alters neutrophil maturation. As such, the term “PMN-MDSC”, if/when correctly used, refers to neutrophils with a distinct identity, deriving from a differentiation reprogramming in response to emergency neutropoiesis signals, expressing a specific transcriptome and phenotype, and intrinsically displaying immunosuppressive properties (lines 515 - 537 and

Table 1). A vast body of literature supporting these and other concepts on PMN-MDSCs exists and cannot be ignored. That is why, for this Perspective, we prefer to keep the PMN-MDSC term. Maintaining a nomenclature that distinguishes mature PMN-MDSCs from conventional neutrophils also reflects a clinical need (as correctly stated by the Reviewer herself/himself). In this context, recent findings described in our Perspective indicate that mPMN-MDSCs exhibit transcriptomic features suggestive of functions beyond immunosuppression (PMID: 38242120; PMID: 40590753), some of which are shared with TANs (PMID: 4059075). Therefore, once the biological properties of mPMN-MDSCs are fully clarified, a revised terminology reflecting their complexity and differences from neutrophil states will facilitate reaching the necessary broad consensus in the field (lines 555 - 559).

That PMN-MDSCs "*represent different types of neutrophils derived from an unknown specific precursor"* is never stated in our article. Similarly, it is never stated that "*all PMN-MDSCs are suppressive*". In fact, as discussed on lines 204 - 215, a major advance in the field has been the demonstration that only the mature PMN-MDSC fraction exhibits suppressive activity (PMID: 28053192; PMID: 29914893; PMID: 32325491). Moreover, as discussed on lines 477 - 480, we recently used scRNA-seq experiments to identify that mature PMN-MDSCs from NSCLC patients comprise a distinct cluster accounting for approximately 50% of the total cells and characterized by immunosuppressive and protumorigenic features (PMID: 40590753). These observations further uncover that even mature PMN-MDSCs may harbor distinct cell populations, some of which are specifically immunosuppressive and protumorigenic.

Overall, the aim and scope of our Perspective were/is to simply discuss current knowledge of human PMN-MDSCs based on recent important discoveries. Discussing *"how neutrophils are regulated at different levels to generate context-dependent and distinct functional pools* or *"how different immunosuppressive neutrophil populations relate to one another in circulation, at steady state",* is out of the scope of this Perspective. In any case, we have incorporated a discussion related to the Reviewer's viewpoint in the Conclusions of the revised Perspective (lines 522 - 531). Moreover, for the Reviewer's info and as requested by the Editor, we have replaced the term "PMN-MDSCs" with "immunosuppressive neutrophils" in the title, while maintaining the original terminology throughout the main text for conceptual and terminological consistency.

Dipartimento di Medicina
Sezione di Patologia Generale
Strada Le Grazie, 8 - 37134 Verona | T: +39 045 8027120
P. IVA 01541040232 | C.F. 93009870234

Dear Marco,

Thank you for submitting the revised version of your perspective to The EMBO Journal. I have now gone through the incoporated rveisions, and I find them reasonable. Therefore, there now remain only a few formatting points that need to be addressed before I can extend the official acceptance of your article.

1. Please provide Table 1 in an editable format.
2. CRediT has replaced the traditional author contributions section because it offers a systematic, machine-readable author contributions format that allows for more effective research assessment. Please remove the Authors Contributions from the manuscript and use the free text boxes beneath each contributing author's name in our online submission system to add specific details on the author's contribution. More information is available in our guide to authors.
3. Please correct the heading of the "Conflict of Interests" section to "Disclosure and Competing Interests Statement".
4. Please check that the funding information is correct and identical both in the manuscript and our online system. The list of funders in our submission system will be linked to PubMed in published articles.

I have forwarded your figures to our collaborating graphic editor Luk Cox from Somersault1824 for final stylistic adaptation. He will be in touch with you directly to discuss the final figures.

I will also look into the manuscript text in detail to see if any adjustments to the journal style are needed.

Please let me know if you have any questions, and I look forward to working with you on the final version of your manuscript!

With best wishes,

Ieva

Ieva Gailite, PhD
Senior Scientific Editor
The EMBO Journal
Meyerhofstrasse 1
D-69117 Heidelberg
Tel: +4962218891309
i.gailite@embojournal.org

Please use the link below to submit your revision:

https://emboj.msubmit.net/cgi-bin/main.plex

All editorial and formatting issues were resolved by the authors.

2nd Revision - Editorial Decision 22nd May 2026

Dear Marco,

Thank you for approving the figures and for implementing the formatting requests. I am now pleased to inform you that your manuscript has been accepted for publication in the EMBO Journal.

Meanwhile, I have gone through your manuscript and added minor edits in the manuscript text file. I would also like to propose to slightly shorten the title to increase its attractiveness for the general readership. Please find the proposed changes in the attached file.

We have also modified the table to include the icons generated by Luk.

Once we have received your approval of these changes, we will be ready for the transfer of your manuscript to our publishers for copy-editing. You will receive page proofs prior to publication.

Should you be planning a Press Release on your article, please notify the production team via this form in order to coordinate publication and release dates: https://forms.cloud.microsoft/e/eH4A8JVP6g

If you have any questions, please do not hesitate to contact the Editorial Office. Thank you for this interesting contribution to The EMBO Journal!

With kind regards,

Ieva

Ieva Gailite, PhD
Senior Scientific Editor
The EMBO Journal
Meyerhofstrasse 1
D-69117 Heidelberg
Tel: +4962218891309
i.gailite@embojournal.org

*****

Please note that it is The EMBO Journal policy for the transcript of the editorial process (containing referee reports and your response letters) to be published as an online supplement to each paper. If you should prefer removal of any referee-only figures included in the point-by-point response(s), e.g. because they may still be used for future publication or because they have been reproduced from published work by others, please do let us know immediately via response email.
More information is available here: https://link.springer.com/partners/embo-press/editorial-policies#Peer%20review

** Click here to be directed to your login page: https://emboj.msubmit.net
